# Supplementary material for: Does Differentially Private Synthetic Data Lead to Synthetic Discoveries?
Source: Methods Inf Med. 2024 Sep 9;63(01-02):35–51. doi: 10.1055/a-2385-1355 (PMC11495942; doi:10.1055/a-2385-1355)
Supplement: Supplementary file 1 — Supplementary Material [file 10-1055-a-2385-1355-s23010044.pdf]

A Supplementary Material

A.1 Drawing data from additively smoothed histograms is differentially private

Proof:

Drawing a single datum from a distribution determined by a histogram can be carried out in a differentially private way using the exponential mechanism, in which the original probabilities are modified as follows. Instead of the relative size of a histogram bin, each possible outcome of the draw has its probability proportional to

$$e^{\frac{\epsilon s_i}{2\Delta}} \ , \tag{1}$$

where s_i is the score of the ith option (e.g. histogram bin) and Δ is the sensitivity of the scoring, indicating the maximum amount the score can change for any outcome if a single datum is changed among the sensitive data. By substituting (1) into (2), it is easy to verify that the exponential mechanism fulfills differential privacy. If we define the score for the ith output as $s_i = \alpha \ln(c_i + \alpha)$, where c_i is the number of sensitive data in the ith bin and $\alpha = \frac{2}{\epsilon}$, the maximal change of the score value takes place when $c_i = 1$ changes to $c_i = 0$ or vice versa. For this maximal change, we have the following upper bound:

$$\begin{aligned}
\alpha \ln(1+\alpha) - \alpha \ln(\alpha) &= \alpha \ln\left(1 + \frac{1}{\alpha}\right) \\
&= \ln\left(\left(1 + \frac{1}{\alpha}\right)^{\alpha}\right) \\
&< \ln(e) \\
&= 1 \ ,
\end{aligned}$$

where the inequality follows from the well-known property of e (see e.g. Heinrich Dörrie [1]):

$$\left(1 + \frac{1}{x}\right)^{x} < e,$$

for all $x > 0$. Thus, the sensitivity of the scoring is 1. Substituting the scoring into (1) indicates that drawing a single datum according to the probabilities proportional to $c_i + \frac{2}{\epsilon}$ is ϵ differentially private.

Due to the sequential composability property, drawing m synthetic data instead of only one datum requires m times larger privacy budget. Consequently, drawing m according to the probabilities proportional to $c_i + \frac{2m}{\epsilon}$ is ϵ differentially private.

[1] Dörrie H. 100 great problems of elementary mathematics: their history and solutions. Dover Publications; 1965

Algorithm 1 Draw synthetic sample

Require: $\boldsymbol{c}$ ▷ Histogram count vector of length h
Require: m ▷ Number of synthetic data to be drawn
Require: ϵ
  $\boldsymbol{s} \leftarrow$ score vector of length h
  $\boldsymbol{p} \leftarrow$ probability vector of length h
  $\boldsymbol{r} \leftarrow$ synthetic data count vector of length h
  $\alpha \leftarrow 2m/\epsilon$ ▷ Calculate amount of additive smoothing
  for $i \in \{1, \dots, h\}$ **do**
    $\boldsymbol{s}_i \leftarrow \alpha \ln(c_i + \alpha)$ ▷ Calculate scores for bins
  for $i \in \{1, \dots, h\}$ **do**
    $p_i \leftarrow e^{\frac{\epsilon s_i}{2m}}$ ▷ Calculate probabilities according to the exponential mechanism
  for $i \in \{1, \dots, h\}$ **do**
    $p_i \leftarrow p_i / \|\boldsymbol{p}\|_1$ ▷ Normalize the probabilities to sum up to one
  $\boldsymbol{r} \leftarrow$ Draw m synthetic data according to the probabilities $\boldsymbol{p}$.
  return $\boldsymbol{r}$

A.2 Failed DP-synthetic data sets

DP-synthetic data generation methods can sometimes produce data sets that fail to meet the requirements of a statistical test, even if the original sensitive data would fulfill these. This type of degenerate behavior was observed in the experiments especially when generating multivariate data for very small ϵ values and small dataset sizes. Typical failure mode observed was that only one class was represented in the synthetic data, in which case none of the tests apply. Further, in some cases all the values of continuous variable were observed to be the same in the synthetic data, in which case t-test is undefined. In the case of the chi-squared test applied to binary variables, highly imbalanced or sparse synthetic data resulted in low expected frequencies, meaning that one or more cells in the contingency table had an expected frequency of less than five, causing the test to fail. In the experiments, Type I and Type II was calculated based on only those generated data sets that met the requirements of the considered tests. Tables S1 and S2 catalogue the number of failed data sets for the experiments performed on the multivariate simulated prostate cancer data.

		Privacy Budget									
		ε = 0.01				ε = 0.1				ε = 1.0	
		Dataset size				Dataset size				Dataset size	
	DP-synthesizer	50	100	500	1000	50	100	500	1000	50	100
MW U test	Private-PGM	211	155	2	1	659	465	32	2	37	0
	MWEM	988	980	630	294	694	330	1	0	2	0
T-test	Private-PGM	343	274	8	4	711	507	36	2	51	1
	MWEM	993	988	655	311	749	360	2	0	3	0
Median test	Private-PGM	278	227	7	4	816	625	58	6	74	1
	MWEM	1000	999	863	570	895	611	9	0	12	0
Chi-Squared test	Private-PGM	347	262	7	2	810	614	69	13	83	17
	MWEM	999	996	829	475	845	514	24	1	40	10

Table S1: Number DP-synthetic datasets that failed to meet the requirements of the statistical test out of 1000 generated for each corresponding DP-synthesizer, privacy budget, and dataset size on simulated multivariate non-signal data. There were no failures in experiments with sample sizes of 20 000, as well as in datasets with sizes of 50, 100, 500, and 100 and $\varepsilon \geq 5$.

		Privacy Budget									
		**ε** = 0.01				**ε** = 0.1				**ε** = 1.0	
		Dataset size				Dataset size				Dataset size	
	DP-synthesizer	50	100	500	1000	50	100	500	1000	50	100
MW U test	Private-PGM	213	156	4	0	647	421	29	2	45	1
	MWEM	986	972	649	354	697	334	3	0	2	0
T-test	Private-PGM	346	265	12	3	686	457	32	3	56	1
	MWEM	990	979	678	367	763	363	5	0	5	0
Median test	Private-PGM	297	206	14	6	788	598	60	6	73	8
	MWEM	1000	998	865	596	877	603	14	0	18	2
Chi-Squared test	Private-PGM	359	260	15	1	793	602	66	9	89	16
	MWEM	1000	994	827	510	848	541	31	6	55	11

Table S2: Number DP-synthetic datasets that failed to meet the requirements of the statistical test out of 1000 generated for each corresponding DP-synthesizer, privacy budget, and dataset size on simulated multivariate signal data. There were no failures in experiments with sample sizes of 20 000, as well as in datasets with sizes of 50, 100, 500, and 100 and $\varepsilon \geq 5$.
